# Social determinants of health and hospital readmissions: can the HOSPITAL risk score be improved by the inclusion of social factors?

**DOI:** 10.1186/s12913-020-05989-7

**Published:** 2021-01-04

**Authors:** Shirlene Obuobi, Rhys F. M. Chua, Stephanie A. Besser, Corey E. Tabit

**Affiliations:** 1 Department of Medicine, University of Chicago, Chicago, IL USA; 2 Section of Cardiology, Department of Medicine, Chicago, IL USA

## Abstract

**Background:**

The HOSPITAL Risk Score (HRS) predicts 30-day hospital readmissions and is internationally validated. Social determinants of health (SDOH) such as low socioeconomic status (SES) affect health outcomes and have been postulated to affect readmission rates. We hypothesized that adding SDOH to the HRS could improve its predictive accuracy.

**Methods:**

Records of 37,105 inpatient admissions at the University of Chicago Medical Center were reviewed. HRS was calculated for each patient. Census tract-level SDOH then were combined with the HRS and the performance of the resultant “Social HRS” was compared against the HRS. Patients then were assigned to 1 of 7 typologies defined by their SDOH and a balanced dataset of 14,235 admissions was sampled from the larger dataset to avoid over-representation by any 1 sociodemographic group. Principal component analysis and multivariable linear regression then were performed to determine the effect of SDOH on the HRS.

**Results:**

The c-statistic for the HRS predicting 30-day readmission was 0.74, consistent with published values. However, the addition of SDOH to the HRS did not improve the c-statistic (0.71). Patients with unfavorable SDOH (no high-school, limited English, crowded housing, disabilities, and age > 65 yrs) had significantly higher HRS (*p* < 0.05 for all). Overall, SDOH explained 0.2% of the HRS.

**Conclusion:**

At an urban tertiary care center, the addition of census tract-level SDOH to the HRS did not improve its predictive power. Rather, the effects of SDOH are already reflected in the HRS.

**Supplementary Information:**

The online version contains supplementary material available at 10.1186/s12913-020-05989-7.

## Background

Hospital readmissions represent a significant expense within the US health system accounting for $17 billion of preventable healthcare costs [1]. The Hospital Readmissions Reduction Program (HRRP) penalizes hospitals with higher excess 30-day readmission rates by reducing Medicare reimbursement. Thus, many groups have published predictive models to identify which patients are at high risk for readmission.

The HOSPITAL Risk Score (HRS), introduced by Donzé et al. in 2013, is a commonly used predictive model for readmissions that has been internationally validated [2, 3]. The score consists of 8 factors which spell “HOSPITAL” acronymically: **H**emoglobin, discharge from an **O**ncology service, **S**odium level at discharge, any coded **P**rocedure during the hospital stay, **I**ndex admission **T**ype, number of previous **A**dmissions in the prior year, and **L**ength of stay. However, the score’s ability to predict readmission risk in the real world has been questioned as the score uses exclusively clinical factors and does not include social determinants of health (SDOH), known contributors to readmission risk [4]. Similarly, when developing metrics for expected readmission rates for individual hospitals, the Centers for Medicare and Medicaid Services (CMS) only included age and gender as non-clinical risk factors for readmission [5].

Authors have recommended adjustment of readmission algorithms to include SDOH to improve their predictive accuracy [6]. By ignoring SDOH in the determination of readmission rates, they argue, CMS may unfairly penalize hospitals that care for the most vulnerable Americans [7]. To ameliorate this, CMS recently adjusted their algorithm to compare readmission rates only between hospitals with similar proportions of low-income patients [8]. This effort resulted in fewer safety-net hospitals being penalized. While a step in the right direction, hospitals who serve patients with unfavorable SDOH still lack a tool to be able to reliably predict which patients are at highest risk for readmission.

The purpose of this study is to assess whether the addition of SDOH to the HRS improved its predictive ability. We hypothesized that the HRS may be improved by integrating more data, specifically pertaining to non-clinical SDOH intrinsic to patients or their communities.

## Methods

### Patient population and study design

We queried a dataset containing all adult patients admitted to our center from 2014 to 2016. As this study measured readmission back to our center, we sought to avoid confounding by excluding patients who lived outside of the city of Chicago, those who were discharged to any location aside from home, and those who lived in very sparsely populated areas of the city.

### Data sources and measures

We calculated the HRS for each patient using data available in our electronic health record (EHR) according to the method described by Donzé [3]. Points were assigned and summed for the following components of the HRS: Hemoglobin < 12 g/dL (1 point), discharge from an Oncology service (2 points), Sodium level at discharge < 135 mEq/L (1 point), any coded Procedure during the hospital stay (1 point), urgent or emergent Index admission Type (1 point), number of previous Admissions in the prior year (0–1, 0 points, 2–5, 2 points, > 5, 5 points), and Length of stay ≥5 days (2 points). Additional variables extracted from the EHR included age, gender, race, ethnicity, laboratory values, vital signs, number of prior readmissions and emergency department (ED) visits, and comorbidities. Fifteen census tract-level SDOH variables which comprise the Social Vulnerability Index (SVI) were obtained from CDC and the census tract-level violent crime rate was obtained from the City of Chicago Data Portal. Neighborhoods then were grouped into sociodemographic clusters using classifications obtained from a published cross-sectional spatial analysis using data from the US Census Bureau [9]. This method provided an objective and evidence-based way to group neighborhoods by common SDOH. The Census block group-level Area Deprivation Index (ADI) was obtained from the University of Wisconsin Neighborhood Atlas [10] and the Census tract-level Hardship Index (HI) was obtained from the City of Chicago Data Portal.

We considered all admissions to our center within the study period as index admissions. Readmissions were defined as any additional admission to our center within 30 days of an index admission. As such, some admissions served as both an index themselves and a readmission for a prior index. The outcome of 30-day readmission was defined dichotomously as the presence or absence of one or more readmissions within 30 days of an index admission.

### Statistical analysis

Continuous variables were represented as median (interquartile ranges) as determined by visualizing the variables, while categorical variables were expressed as frequencies and percentages. A Spearman rank correlation test was completed to assess for the multicollinearity of clinical and social variables (Fig. S1). Since social variables were the variables of interest for the study and they showed multicollinearity, they were grouped into components using Principal Component Analysis (PCA). Cronbach alpha tests were performed to confirm the internal consistency of each component; each component had a Cronbach alpha value above 0.57. Bartlett’s test of sphericity (*p* < 0.001) and Kaiser-Meyer-Olkin (KMO) sampling adequacy (MSA = 0.85) were conducted to further confirm adequate sample size and correlation between the social variables. Variables included in the PCA analysis were percentages for poverty, unemployment, per capita income, disability, single parent households, minority, no vehicle, no high school diploma, age 65 years and above, limited English, crowding, multiunit living, and violent crime rate. After scaling the data, PCA with varimax rotation was performed on unsampled, randomly sampled, gender-stratified, and disease-stratified datasets. Scree plot was used to determine the optimal number of components needed to explain the total variance. A four component PCA solution containing social variables with a cutoff of PCA loading of 0.62 was found to explain 80% of the total variance. In addition, a parallel analysis was used to confirm the use of a four component PCA. Spearman correlation analysis was performed on the components to confirm their independence, and then based upon the items in each of the components, they were named the following categories: low income, no high school diploma, no vehicle and multiunit living, and age 65 years and above or disabled.

Since most patients at our center reside in the extreme poverty and suburban affluent clusters, we randomly sampled 600 patients from each of those clusters to create a balanced dataset. A multivariable linear regression was used to test for significance against the HOSPITAL score with low income, no high school diploma, no vehicle and multiunit living, age 65 years and above or disabled, congestive heart failure (CHF), valvular heart diseases, hypertension, diabetes mellitus, renal diseases, liver diseases, chronic obstructive pulmonary disease (COPD), atrial fibrillation (AF), dyslipidemia, and coronary artery disease as independent variables derived from PCA analysis. Receiver Operating Curve analysis (ROC) was used to determine the c-statistic for models with HRS and social variables and HRS without social variables. Statistical significance was defined as a *p*-value < 0.05 for two-tailed tests. Data were analyzed using RStudio version 3.5.1 (RStudio: Integrated Development for R, RStudio, Inc. 2015, Boston, MA). Statistical models were performed using these packages in R: psych (version 2.0.7), corrplot (version 0.84), FactoMineR (version 2.3), and ade4 (version 1.7).

Multiple sensitivity analyses were performed by repeating our analysis after replacing the SVI with the HI and again with the ADI. Variables within the HI include crowding, poverty, unemployed and age 16 and above, no high school diploma and age 25 and above, age 18 and under and 64 and above, and per capita income. A two component PCA solution containing social variables with a cutoff of PCA loading of 0.6 was found to explain 83% of the total variance. Based upon the variables in each component, they were named low income and no high school diploma. A multivariable linear regression was used to test for significance against the HOSPITAL score using low income, no high school diploma, CHF, valvular diseases, hypertension, diabetes mellitus, renal diseases, liver diseases, COPD, AF, dyslipidemia, and coronary artery disease as independent variables. ROC was used to determine the c statistic for each model.

The ADI was used to perform a multivariable linear regression and was used to test for significance against HOSPITAL score using the state ADI ranking, CHF, valvular diseases, hypertension, diabetes mellitus, renal diseases, liver diseases, COPD, AF, dyslipidemia, and coronary artery disease as independent variables. ROC was used to determine the c statistic for each model.

## Results

### Participant characteristics

**Fig. 1:**
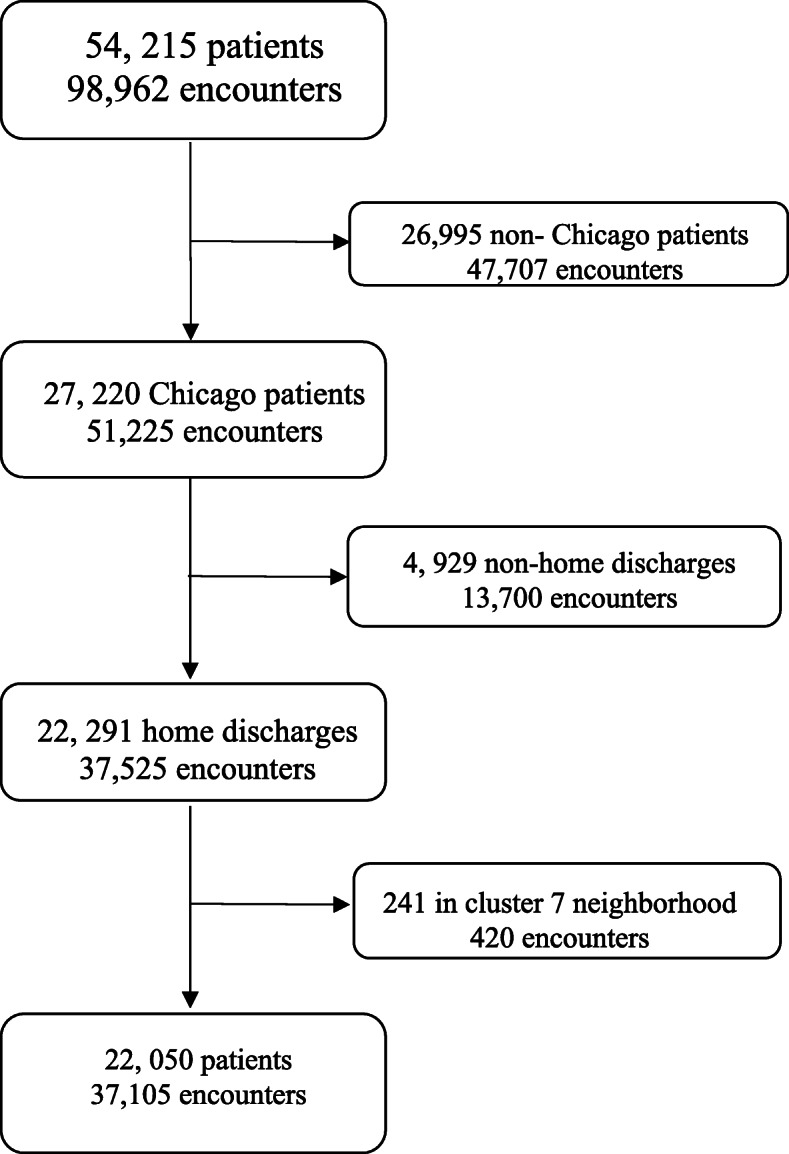
Patient Inclusion: Schema depicting inclusion and exclusion criteria for our study. All patients treated at our center 2014–2016 were queried. Patients living outside Chicago, patients discharged to any place other than home, and patients living in very sparsely populated areas of the city were excluded

A total of 54,215 records were queried and 37,105 participants met the inclusion criteria (Fig. 1). Median age of patients was 53 years (IQR 33–67 years). The majority of patients were female (63.8%), African-American (80.3%), not Hispanic or Latino (94.0%), and resided in the extreme poverty cluster (63.2%). The median household income was $32,401 (IQR $27,091 - $40,587). Clinical and social variables are summarized in Table 1.

**Table 1:** Participant Characteristics

**Demographics**	
Age (years), median [IQR]	53.00 [33.00, 67.00]
Gender, n (%)	
Male	13,427 (36.2)
Female	23,678 (63.8)
Race, n (%)	
Black/African-American	29,797 (80.3)
White	5424 (14.6)
Other	1884 (5.1)
Ethnicity, n (%)	
Hispanic or Latino	1758 (4.7)
Not Hispanic or Latino	34,871 (94.0)
Unknown	476 (1.3)
**Vital Signs, median [IQR]**	
Systolic blood pressure, mmHg	123.00 [110.00, 137.00]
Diastolic blood pressure, mmHg	70.00 [61.00, 79.00]
Heart rate, beats per minute	80.00 [71.00, 90.00]
BMI	27.46 [23.19, 33.30]
**Laboratories, median [IQR]**	
Hemoglobin, g/dL	10.60 [9.30, 12.10]
Hematocrit, %	32.30 [28.40, 36.50]
Platelets, 10*3/uL	226.00 [178.00, 287.00]
Sodium, mEq/L	138.00 [136.00, 140.00]
Potassium, mEq/L	4.00 [3.70, 4.30]
BUN, mg/dL	13.00 [9.00, 21.00]
Creatinine, mg/dL	0.90 [0.70, 1.20]
NT-ProBNP, pg/mL	1535.50 [266.25, 6024.50]
**Comorbidities, n (%)**	
Heart Failure	6050 (16.3)
Coronary Artery Disease	5784 (15.6)
Atrial Fibrillation	2786 (7.5)
Hypertension	18,421 (49.6)
Dyslipidemia	6731 (18.1)
Chronic Obstructive Pulmonary Disease	3616 (9.7)
Diabetes	8569 (23.1)
Valvular Heart Disease	2692 (8.5)
Renal Disease	7203 (22.8)
Liver Disease	2429 (7.7)
Medications, n (%)	
Antiplatelets	10,285 (27.7)
Beta Blockers	12,233 (33.0)
ACE/ARB	10,919 (29.4)
Aldosterone Antagonist	1953 (5.3)
Loop Diuretic	6422 (17.3)
Statins	11,195 (30.2)
Anticoagulants	23,080 (62.2)
Insulin	9241 (24.9)
HOSPITAL risk score, n (%)	
Low risk (≤ 4)	22,223 (71.1)
Intermediate risk (5, 6)	5648 (18.1)
High risk (≥ 7)	3364 (10.8)
Prior Health System Utilization, n (%)	
Number of readmissions 1 month before index	0.18 (0.47)
Number of ER visits 1 month before index	0.25 (0.66)
Social Vulnerability Index Components, median [IQR]	
Age 65 and above (% of census tract)	13.80 [9.40, 17.30]
Crowding (% of census tract)	3.00 [0.90, 5.20]
Disabled (% of census tract)	14.30 [10.90, 17.80]
Limited English (% of census tract)	0.60 [0.00, 2.20]
Minority (% of census tract)	98.00 [89.90, 99.50]
Mobile home (% of census tract)	0.00 [0.00, 0.00]
No high school diploma (% of census tract)	13.90 [9.90, 19.80]
No vehicle (% of census tract)	34.80 [24.50, 43.00]
Per capita income ($)	19,064.00 [14,715.00, 25,467.00]
Poverty (% of census tract)	30.00 [19.90, 39.30]
Single parent household (% of census tract)	15.20 [9.70, 20.80]
Unemployment (% of census tract)	19.20 [12.20, 25.90]
Hardship Index Components, median [IQR]	
Crowding (% of census tract)	3.30 [2.40, 4.00]
Poverty (% of census tract)	29.00 [19.20, 30.70]
Aged 16+ and unemployed (% of census tract)	20.30 [15.70, 24.00]
Aged 25+ and no high school diploma (% of census tract)	16.50 [14.00, 21.00]
Aged under 18 and over 64 (% of census tract)	39.50 [35.70, 41.10]
Per capita income ($)	18,672.00 [16,563.00, 23,791.00]
Neighborhood cluster type, n (%)	
Rural Affordable	2516 (6.8)
Vibrant Urban Core	1502 (4.0)
Suburban Affordable	941 (2.5)
Extreme Poverty	23,461 (63.2)
Multilingual Working	1276 (3.4)
Suburban Affluent	7409 (20.0)
Violent crime rate (% of census tract)	48.87 [25.55, 71.76]
Area Deprivation Index Score	7.00 [5.00, 9.00]

### Outcomes

**Fig. 2:**
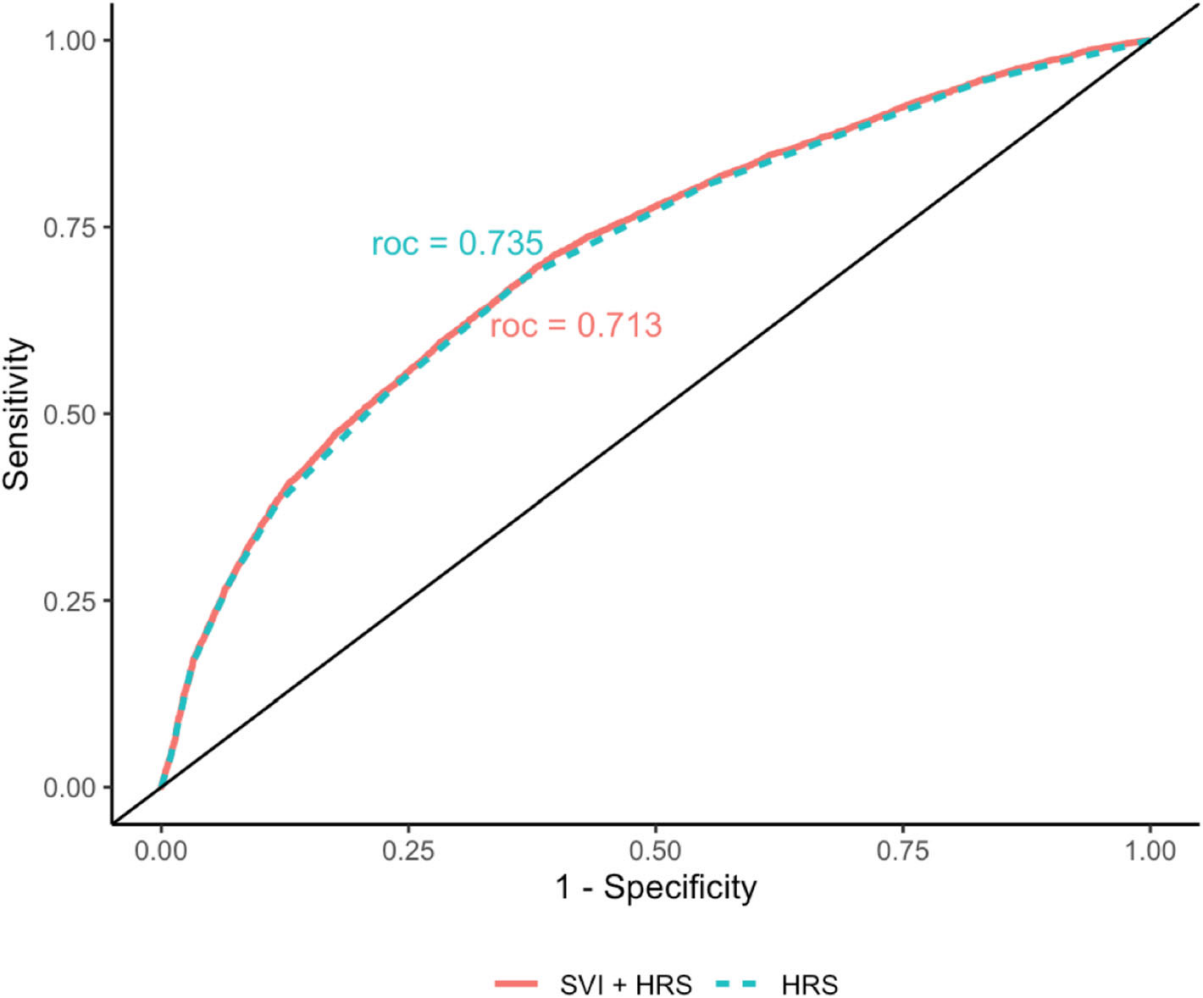
ROC Analysis for HRS and Social HRS: Addition of SVI to the HRS did not improve predictive performance

The c-statistic for the HRS predicting 30-day readmission in our dataset was 0.735 which is similar to the published value (0.72) [2]. However, the addition of SDOH to create the “social HRS” did not improve the predictive power (c-statistic = 0.713, Fig. 2). This finding persisted in the balanced dataset as well (0.721) suggesting that over-representation of patients living in extreme poverty at our center was not the cause of the negative results. This finding further persisted when patients were stratified by presence or absence of a recent admission within 30 days prior to the index admission (Fig. S2a-b) and when the ADI or HI were substituted for the SVI (Fig. S3a-b). Rather, several SDOH, including patients with no high school diploma (β = 0.062, *p* < 0.001), no vehicle and multiunit living (β = − 0.060, *p* < 0.001), CHF (β = 0.142, *p* < 0.001), valvular disease ((β = 0.480, *p* < 0.001), diabetes mellitus (β = 0.093, *p* < 0.005), renal disease (β = 0.740, *p <* 0.001), liver disease (β = 0.688, *p <* 0.001), COPD (β = 0.345, *p <* 0.001), AF (β = 0.169, *p <* 0.001), and dyslipidemia (β = − 0.278, *p <* 0.001) were significantly associated with higher HRS scores. PCA component scores are shown in Tables S1 through S12.

Using components from PCA in three separate subanalyses, we found that patients who were disabled or over 65 years of age had a higher HRS than those who were younger and not disabled if they had coronary artery disease (β = 0.31, *p* < 0.001), liver disease (β = 0.32, *p* < 0.05), or pulmonary disease (β = 0.18, *p <* 0.001). Similarly, low-income patients with cardiac valvular disease (β = 0.37, *p* < 0.005), and obesity (β = 0.12, *p* < 0.05) also had a higher HRS than similar higher earners. Among females, those who were low income (β = 0.037, *p <* 0.05), those with no vehicle and living in multiunit housing (β = − 0.033, *p <* 0.05), and those with CHF (β = 0.136, *p* < 0.005), valvular disease (β = 0.493, *p* < 0.001), renal disease (β = 0.819, *p <* 0.001), liver disease (β = 0.619, *p <* 0.001), COPD (β = 0.251, *p* < 0.001), or dyslipidemia (β = − 0.262, *p* < 0.001) had a higher HRS. Linear regression estimates are shown in Table S13-S24. Overall, SDOH explained 0.2% of the HRS.

Analysis using HI showed that patients with no high school diploma (β = 0.069, *p* < 0.001), CHF (β = 0.139, *p* < 0.001), valvular disease (β = 0.481, *p <* 0.001), diabetes mellitus (β = 0.096, *p <* 0.005), renal disease (β = 0.1740 *p <* 0.001), liver disease (β = 0.697, *p <* 0.001), COPD (β = 0.399, *p <* 0.001), AF (β = 0.163, *p <* 0.001), and dyslipidemia (β = − 0.277, *p <* 0.001) had a higher HRS score. In another subanalyses, low income patients had a higher HRS if they were obese (β = 0.129, *p <* 0.005) and had valvular heart disease (β = 0.372, *p <* 0.005). Among females, those who were low income (β = 0.025, *p <* 0.05), those with no high school diploma (β = 0.041, *p <* 0.005), and those with valvular disease (β = 0.4935 *p <* 0.001), diabetes mellitus (β = 0.045, *p <* 0.001), renal disease (β = 0.819, *p <* 0.001), liver disease (β = 0.620, *p <* 0.001), COPD (β = 0.250, *p <* 0.001), or dyslipidemia (β = − 0.265, *p <* 0.001) had a higher HRS. PCA component scores for HI are shown in Tables S25-S36. Linear regression estimates are shown in Table S37-S48. Similar to the analysis using SDOH, HI explained 0.2% of the HRS.

## Discussion

In this study, we sought to determine if the predictive performance of the HRS could be improved by integrating SDOH into its structure (Social HRS). Surprisingly, we found that adding SDOH as variables did not improve the HRS’ performance. Rather, it appears that patients with poor SDOH are clinically more ill and this increased illness is already captured in the HRS.

In support of this conclusion, we found that patients who had both unfavorable SDOH such as older age, disability status, low SES, without vehicles, and who are in multiunit living, and chronic diseases such as CAD, liver disease, and pulmonary disease had significantly higher HRS. These conditions have high morbidity and mortality at baseline, both of which may be exacerbated by unfavorable SDOH, leading to more frequent readmissions. However, even in these populations, SDOH only explained 0.2% of the HRS. SDOH, by definition, are independently associated with health outcomes and life expectancy [11]. Patients with unfavorable SDOH tend to have more chronic medical conditions and present to the hospital with more advanced disease [12, 13]. Thus, the clinical factors included in the HOSPITAL score, such as hemoglobin, number of admissions in the last year, and length of stay, likely already reflect the effects of SDOH. Therefore, addition of SDOH to HRS does not appear to improve its predictive power.

These findings are consistent with a study by Bernheim et al. in which adjusting for SES did not affect estimated readmission rates [14]. Similarly, a study out of Ontario found no link between SES and readmission [15]. Our study builds on this prior work by demonstrating for the first time that the HRS is objectively higher in patients with poor SDOH and that addition of SDOH to the HRS is not necessary for predictive accuracy.

Notably, programs such as the Coalition project that attempted to reduce admissions among high utilizers with interventions targeting SDOH have had limited impact on readmission rates [16]. These results were obtained in the context of a universal health care system, which may have mitigated issues with access to healthcare. While there are disparities in healthcare access in the United States and within the population our institution serves, our study was specifically focused on patients who were admitted to the hospital and therefore did have access to healthcare. Further analysis could include patients without insurance or otherwise less access to healthcare.

These results do not imply that SDOH do not influence readmission rates. Multiple studies have demonstrated that SDOH such as race, socioeconomic status, and education contribute to a higher risk of readmission [17–20]. A study by Barnett et al. found that half of the difference in readmission rates between hospitals with highest and lowest rates of readmission could be explained by patient characteristics outside of the hospital’s control [6]. Additionally, a metanalysis by Van Walraven et al. found that predictive models for readmission that included SDOH in their algorithms were able to identify twice as many avoidable readmissions as those that used only clinical factors [18, 21]. These models have been found to be weaker when applied to patient populations with poor SDOH, which potentially makes models like the HRS less useful in safety-net hospitals [22].

The mechanisms by which SDOH influence readmissions are complex and difficult to define. For example, for the cross-section of unmarried men with low incomes, Social HRS was lower than HRS. Thus, even though having a low SES is considered an unfavorable SDOH, within this intersection, patients were less likely to be readmitted within 30 days. This may be because unmarried men are less likely to interface with doctors. The 2017 MENtion it Survey by Cleveland Clinic showed that only 61% of men go to their doctor even after developing symptoms that they describe as “unbearable,” and that 83% of married women remind their husbands to attend annual checkups [23]. A qualitative study that interviewed physicians at seven hospitals with high readmissions rates found that most physicians asserted that readmissions were influenced by factors such as patient trust and willingness to participate as well as other social factors [24]. Additional patient attributes such as social support and personal resilience factors such as patient adaptability and biologic stress mechanisms also influence disease severity, which in turn influences readmission rates [25].

Our study has several limitations. First, we utilized census tract-level SDOH in this analysis. Individual-level SDOH are influenced but not entirely explained by neighborhood factors. Patient-level data may more accurately encapsulate resilience factors and lead to a different conclusion. The SVI was used in this manuscript because it is easily available at the census-tract level, well validated, and included in other community-level tools. Our findings were also similar when the ADI or HI were substituted for the SVI. The authors acknowledge that other factors such as legal status and environmental factors may alter the results and we believe further studies exploring these factors’ potential contribution to readmission risk should be undertaken.

Additionally, participants were studied at a single tertiary care center that serves a large population of urban poor as well as patients with advanced illnesses. Patients seen at our institution who have more favorable SDOH likely traveled a longer distance to our center and may have been self-selected due to the severity of their illness. These patients may have been on a trajectory toward frequent readmissions and similarly would have a higher HRS. To address this, we sampled a balanced dataset and found similar results. However, our dataset remains bereft of patients outside of a metropolitan area and would likely not be generalizable to hospitals that serve more rural populations. This could be an area for further research.

While we have tested for multicollinearity among variables, correlation of two variables does not equate to a linear combination of the vector space and linear dependence is rarely influenced by two dimensions alone. Correlation of two variables does not provide information about the relative importance of each variable. The authors acknowledge these limitations of our models. This study is further limited by the lookback period length (30 days). While similar results were obtained when the analysis was stratified by the presence or absence of an admission in the prior 30 days, it is possible that other lookback period lengths may produce different results.

Finally, this study examined patients admitted to our center and readmitted back to our center. We were not able to determine if patients were admitted to a different center and then readmitted here, or admitted here and then readmitted elsewhere. However, we have previously found that 95% of patients discharged from our center who require readmission are readmitted back to our center with only 5% readmitted elsewhere [26]. This ratio has been stable for many years at our center, including the time of the present study.

## Conclusion

The addition of SDOH does not improve the predictive accuracy of the HRS. Rather, the effects of unfavorable SDOH manifest as overall worse health which is already captured in the HRS.

## Supplementary Information

**Additional file 1: Fig. S1.** Correlation plot of HRS components and SDOH: Components of the HRS showed minimal collinearity with SDOH. **Fig. S2.** ROC for patients with and without admission 30 days before index: When stratified by the (a) presence or (b) absence of a prior admission within the prior 30 days, the addition of SDOH to the HRS did not improve its performance, similar to the unstratified dataset. **Fig. S3.** ROC for HRS and ADI + HRS or HRS and HI + HRS: Repeating our analysis by substituting the (a) ADI or (b) HI for the SVI produced similar results to our initial analyses; the addition of measures of SDOH did not improve the predictive performance of the HRS. **Table S1.** PCA Component Scores, all patients. **Table S2.** PCA Component Scores, randomly-sampled balanced dataset. **Table S3.** PCA Component Scores, patients with heart failure. **Table S4.** PCA Component Scores, patients with atrial fibrillation. **Table S5.** PCA Component Scores, patients with coronary artery disease. **Table S6.** PCA Component Scores, patients with COPD. **Table S7.** PCA Component Scores, patients with liver disease. **Table S8.** PCA Component Scores, patients with obesity. **Table S9.** PCA Component Scores, patients with pulmonary disease. **Table S10.** PCA Component Scores, patients with valvular heart disease. **Table S11.** PCA Component Scores, female patients. **Table S12.** PCA Component Scores, male patients. **Table S13.** Linear Regression Estimates, all patients. **Table S14.** Linear Regression Estimates, randomly-sampled balanced dataset. **Table S15.** Linear Regression Estimates, patients with heart failure. **Table S16.** Linear Regression Estimates, patients with atrial fibrillation. **Table S17.** Linear Regression Estimates, patients with coronary artery disease. **Table S18.** Linear Regression Estimates, patients with COPD. **Table S19.** Linear Regression Estimates, patients with liver disease. **Table S20.** Linear Regression Estimates, patients with obesity. **Table S21.** Linear Regression Estimates, patients with pulmonary disease. **Table S22.** Linear Regression Estimates, patients with valvular heart disease. **Table S23.** Linear Regression Estimates, female patients. **Table S24.** Linear Regression Estimates, male patients. **Table S25.** PCA Component Scores, all patients. **Table S26.** PCA Component Scores, randomly-sampled balanced dataset. **Table S27.** PCA Component Scores, patients with heart failure. **Table S28.** PCA Component Scores, patients with atrial fibrillation. **Table S29.** PCA Component Scores, patients with coronary artery disease. **Table S30.** PCA Component Scores, patients with COPD. **Table S31.** PCA Component Scores, patients with liver diseases. **Table S32.** PCA Component Scores, patients with obesity. **Table S33.** PCA Component Scores, patients with pulmonary disease. **Table S34.** PCA Component Scores, patients with valvular heart disease. **Table S35.** PCA Component Scores, female patients. **Table S36.** PCA Component Scores, male patients. **Table S37.** Linear Regression Estimates, all patients. **Table S38.** PCA Component Scores, randomly-sampled balanced dataset. **Table S39.** PCA Component Scores, patients with heart failure. **Table S40.** PCA Component Scores, patients with atrial fibrillation. **Table S41.** PCA Component Scores, patients with coronary artery disease. **Table S42.** PCA Component Scores, patients with COPD. **Table S43.** PCA Component Scores, patients with liver disease. **Table S44.** PCA Component Scores, patients with obesity. **Table S45.** PCA Component Scores, patients with pulmonary disease. **Table S46.** PCA Component Scores, patients with valvular heart disease. **Table S47.** Linear Regression Estimates, female patients. **Table S48.** Linear Regression Estimates, male patients.

## Data Availability

Interested parties wishing to obtain the data used in the present study may contact the communicating author. Data will only be released with the approval of the IRB at the University of Chicago.

## Abbreviations

ADI: Area Deprivation Index
AF: Atrial Fibrillation
CAD: Coronary Artery Disease
CHF: Congestive Heart Failure
CMS: Centers for Medicare and Medicaid Services
COPD: Chronic Obstructive Pulmonary Disease
EHR: Electronic Health Record
HI: Hardship Index
HRRP: Hospital Readmissions Reduction Program
HRS: HOSPITAL Risk Score
KMO: Kaiser-Meyer-Olkin
PCA: Principal Component Analysis
ROC: Receiver Operating Curve
SDOH: Social Determinants of Health
SES: Socioeconomic Status
SVI: Social Vulnerability Index

## References

[1] Centers for Medicare and Medicaid S. Guide to Reducing Disparities in Readmissions Guide to Reducing Disparities in Readmissions Acknowledgments: Centers for Medicare and Medicaid Services; 2018.

[2] Donze JD, Williams MV, Robinson EJ (2016). International validity of the HOSPITAL score to predict 30-day potentially avoidable hospital readmissions. JAMA Intern Med.

[3] Donze J, Aujesky D, Williams D, Schnipper JL (2013). Potentially avoidable 30-day hospital readmissions in medical patients: derivation and validation of a prediction model. JAMA Intern Med.

[4] Nguyen OK, Halm EA, Makam AN (2016). Further limitations of the HOSPITAL score in US hospitals. Am Med Assoc.

[5] McIlvennan CK, Eapen ZJ, Allen LA (2015). Hospital readmissions reduction program. Circulation..

[6] Barnett ML, Hsu J, McWilliams JM (2015). Patient characteristics and differences in hospital readmission rates. JAMA Intern Med.

[7] Gilman M, Adams EK, Hockenberry JM, Wilson IB, Milstein AS, Becker ER (2014). California safety-net hospitals likely to be penalized by ACA value, readmission, and meaningful-use programs. Health Aff.

[8] Rau J (2018). Medicare eases readmission penalties against safety-net hospitals.

[9] Kolak M, Bhatt J, Park YH, Padron NA, Molefe A (2020). Quantification of neighborhood-level social determinants of health in the continental United States. JAMA Netw Open.

[10] University of Wisconsin School of Medicine and Publich Health. Area Deprivation Index v2.0. . Accessed Downloaded from https://www.neighborhoodatlas.medicine.wisc.edu/ October 19, 2020.

[11] Marmot M (2005). Social determinants of health inequalities. Lancet..

[12] Mensah GA, Mokdad AH, Ford ES, Greenlund KJ, Croft JB (2005). State of disparities in cardiovascular health in the United States.

[13] Center for Health Analytics RaTaN (2019). Eroding the Fabric of a Healthy Society.

[14] Bernheim SM, Parzynski CS, Horwitz L (2016). Accounting for patients’ socioeconomic status does not change hospital readmission rates. Health Aff.

[15] van Walraven C, Wong J, Forster AJ (2013). Influence of neighborhood household income on early death or urgent hospital readmission. J Hosp Med.

[16] Finkelstein A, Zhou A, Taubman S, Doyle J (2020). Health care Hotspotting — a randomized, controlled trial. N Engl J Med.

[17] Weissman JS, Stern RS, Epstein AM (1994). Prospective Study in Four Massachusetts Hospitals The Impact of Patient Socioeconomic and Other Social Factors on Readmission : A Prospective Study in Hospitals.

[18] Joynt KE, Jha AK (2012). Thirty-day readmissions - Truth and consequences. Massachussetts Med Soc.

[19] Schwarz KA, Elman CS (2003). Identification of factors predictive of hospital readmissions for patients with heart failure. Heart Lung.

[20] Roberts ET, Zaslavsky AM, Barnett ML, Landon BE, Ding L, McWilliams JM (2018). Assessment of the effect of adjustment for patient characteristics on hospital readmission rates: implications for pay for performance. JAMA Intern Med.

[21] van Walraven C, Bennett C, Jennings A, Austin PC, Forster AJ. Proportion of hospital readmissions deemed avoidable: a systematic review. CMAJ. 2011;183(7):E391–402. 10.1503/cmaj.101860. 10.1503/cmaj.101860 PMC3080556 21444623

[22] Morgan DJ, Bame B, Zimand P (2019). Assessment of machine learning vs standard prediction rules for predicting hospital readmissions. JAMA Netw Open.

[23] Cleveland Clinic MENtion it Survey Results*.*.

[24] Joynt KE, Sarma N, Epstein AM, Jha AK, Weissman JS (2014). Challenges in reducing readmissions: lessons from leadership and frontline personnel at eight minority-serving hospitals. Jt Comm J Qual Patient Saf.

[25] Palmer RC, Ismond D, Rodriquez EJ, Kaufman JS (2019). Social determinants of health: future directions for health disparities research. Am J Public Health.

[26] Tabit CE, Coplan MJ, Spencer KT, Alcain CF, Spiegel T, Vohra AS, Adelman D, Liao JK, Sanghani RM. Cardiology Consultation in the Emergency Department Reduces Re-hospitalizations for Low-Socioeconomic Patients with Acute Decompensated Heart Failure. Am J Med. 2017;130(9):1112.e17–1112.e31. 10.1016/j.amjmed.2017.03.044. 10.1016/j.amjmed.2017.03.044 PMC5572103 28457798

