# Supplementary material for: Social determinants of health and hospital readmissions: can the HOSPITAL risk score be improved by the inclusion of social factors?
Source: BMC Health Serv Res. 2021 Jan 4;21:5. doi: 10.1186/s12913-020-05989-7 (PMC7780407; doi:10.1186/s12913-020-05989-7)
Supplement: Supplementary file 1 — Additional file 1: Fig. S1. Correlation plot of HRS components and SDOH: Components of the HRS showed minimal collinearity with SDOH. Fig. S2. ROC for patients with and without admission 30 days before index: When stratified by the (a) presence or (b) absence of a prior admission within the prior 30 days, the addition of SDOH to the HRS did not improve its performance, similar to the unstratified dataset. Fig. S3. ROC for HRS and ADI + HRS or HRS and HI + HRS: Repeating our analysis by substituting the (a) ADI or (b) HI for the SVI produced similar results to our initial analyses; the addition of measures of SDOH did not improve the predictive performance of the HRS. Table S1. PCA Component Scores, all patients. Table S2. PCA Component Scores, randomly-sampled balanced dataset. Table S3. PCA Component Scores, patients with heart failure. Table S4. PCA Component Scores, patients with atrial fibrillation. Table S5. PCA Component Scores, patients with coronary artery disease. Table S6. PCA Component Scores, patients with COPD. Table S7. PCA Component Scores, patients with liver disease. Table S8. PCA Component Scores, patients with obesity. Table S9. PCA Component Scores, patients with pulmonary disease. Table S10. PCA Component Scores, patients with valvular heart disease. Table S11. PCA Component Scores, female patients. Table S12. PCA Component Scores, male patients. Table S13. Linear Regression Estimates, all patients. Table S14. Linear Regression Estimates, randomly-sampled balanced dataset. Table S15. Linear Regression Estimates, patients with heart failure. Table S16. Linear Regression Estimates, patients with atrial fibrillation. Table S17. Linear Regression Estimates, patients with coronary artery disease. Table S18. Linear Regression Estimates, patients with COPD. Table S19. Linear Regression Estimates, patients with liver disease. Table S20. Linear Regression Estimates, patients with obesity. Table S21. Linear Regression Estimates, patients with pulmonary di [file 12913_2020_5989_MOESM1_ESM.docx]

**Supplemental Figures**


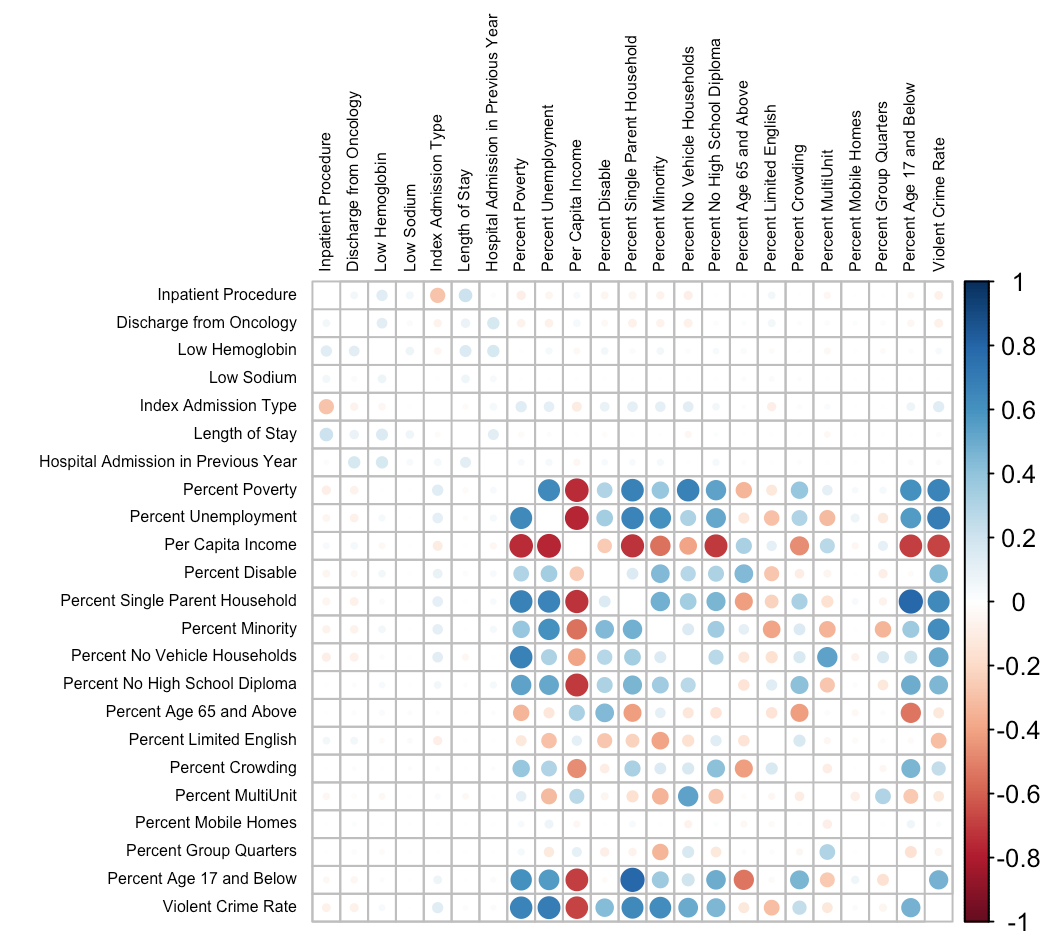
**Figure S1 – Correlation plot of HRS components and SDOH**

**
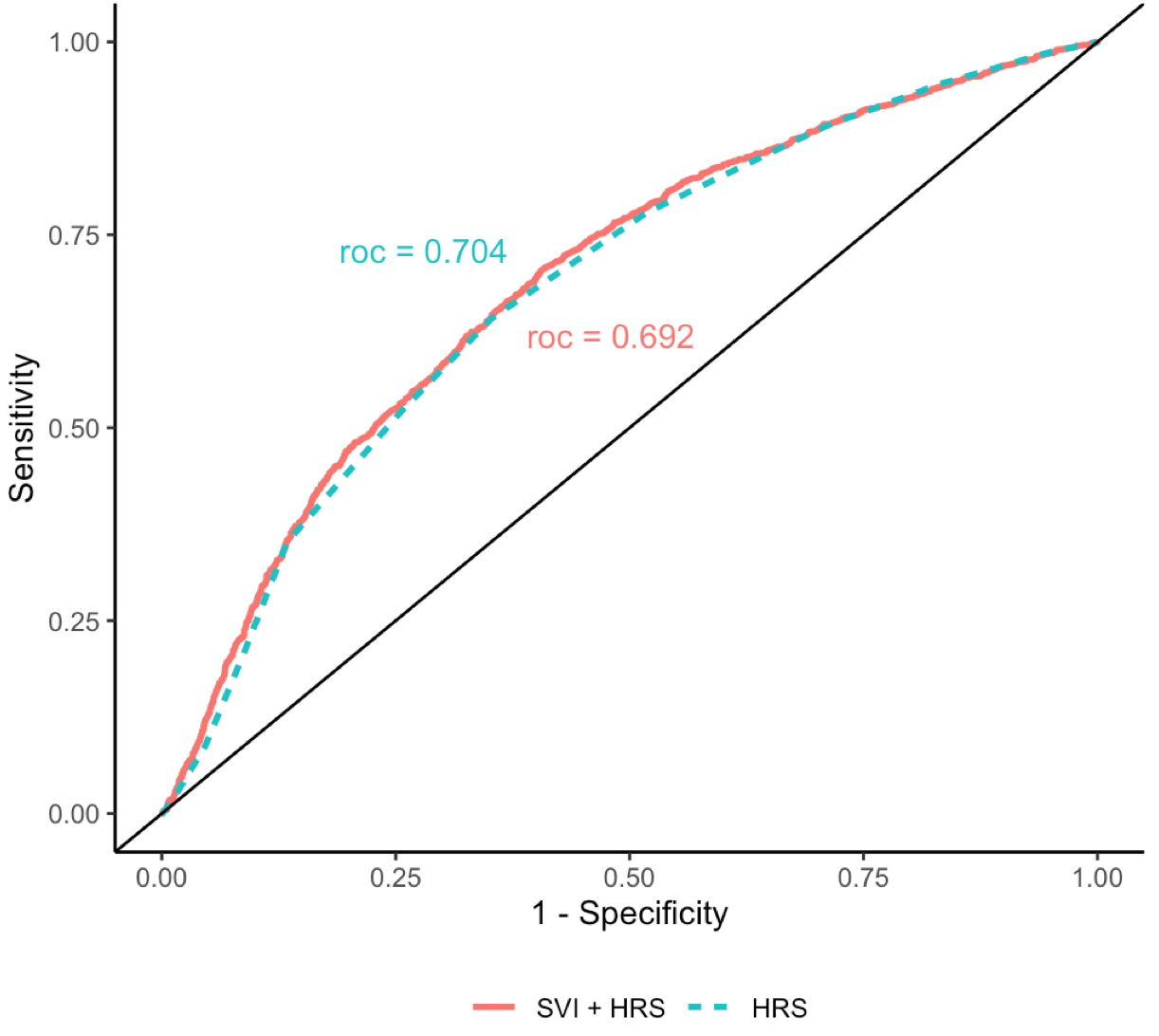
Figure S2a – ROC for patients with admission 30 days before index**

**
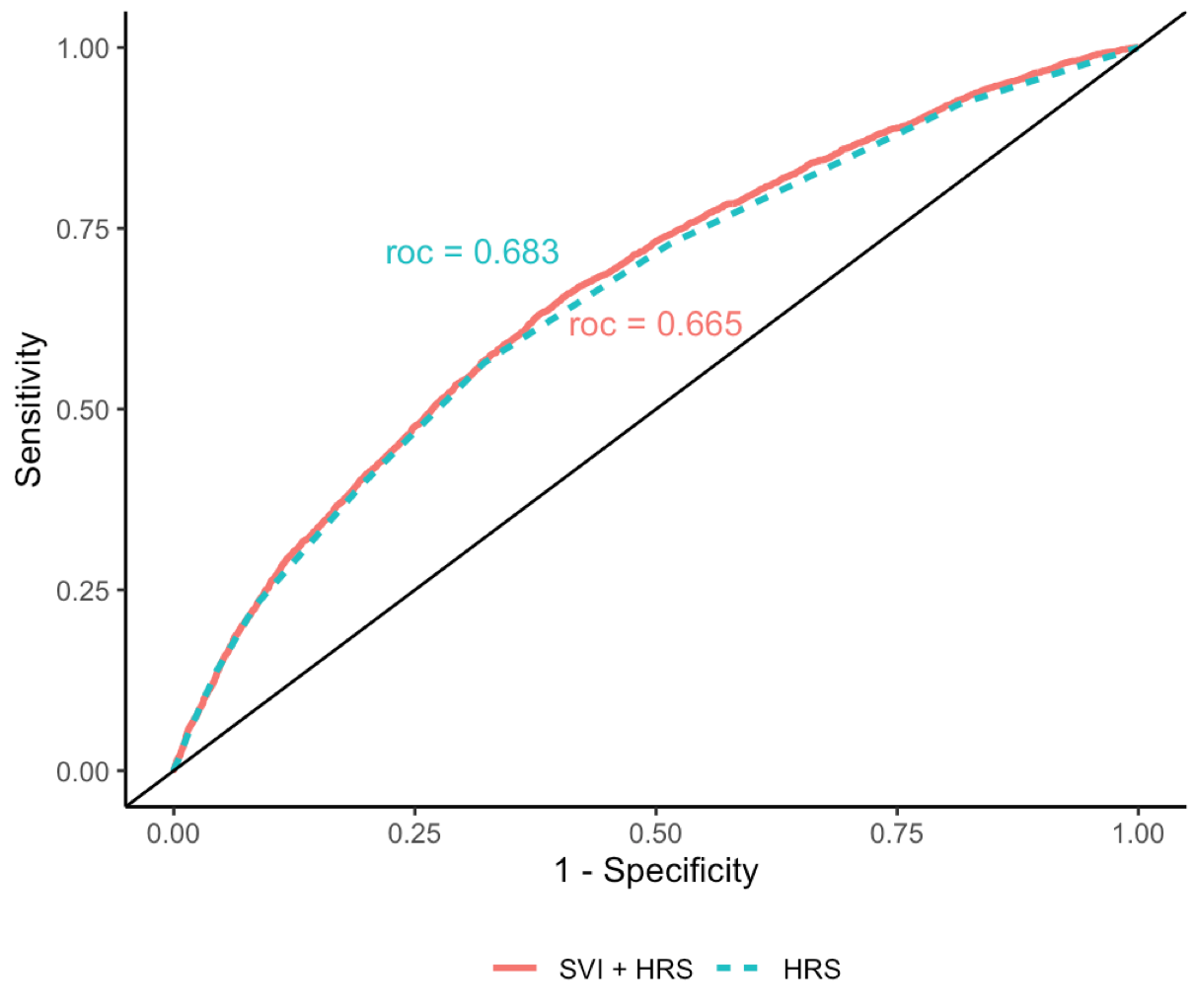
Figure S2b - ROC for patients without admission 30 days before index**

**
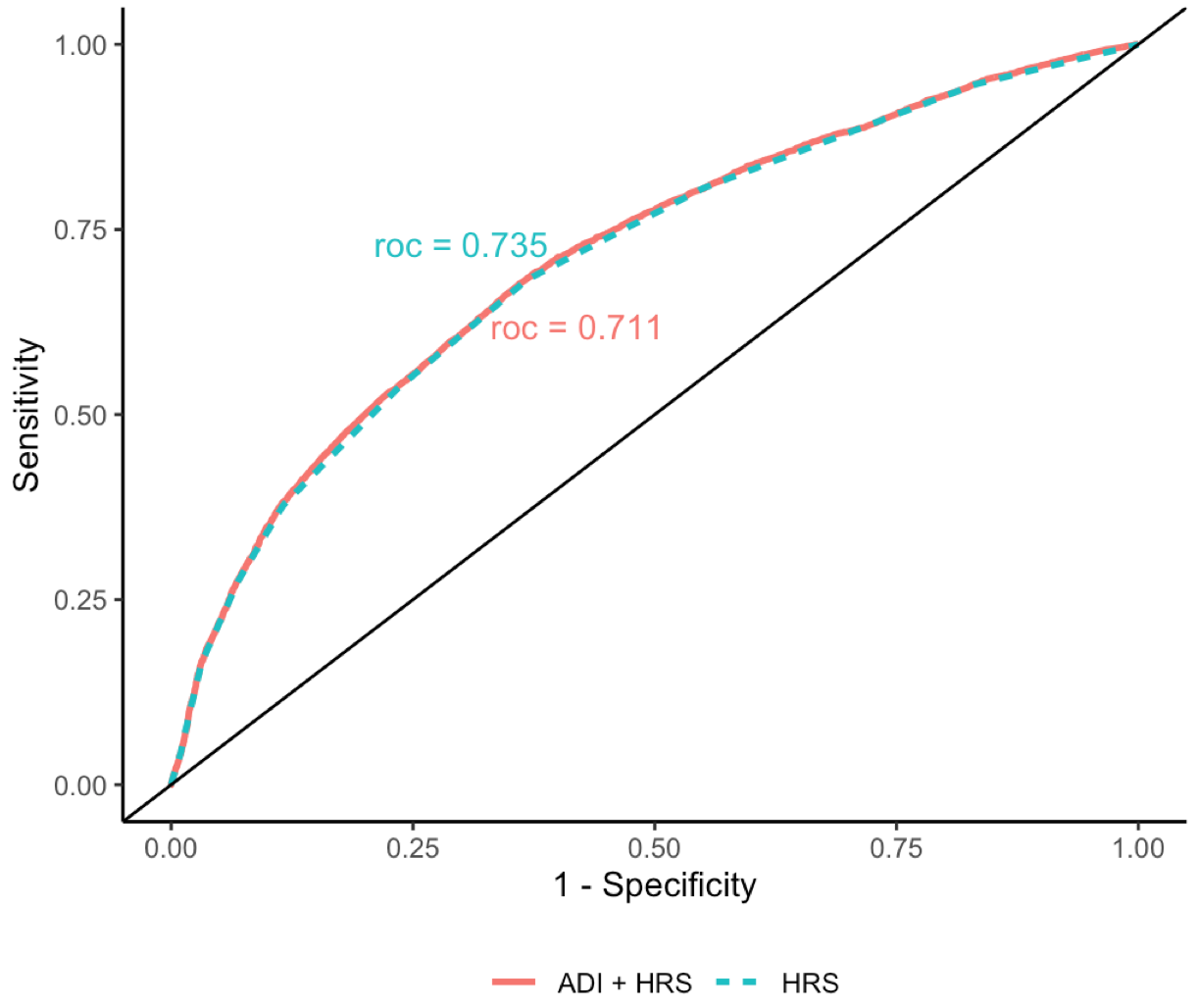
Figure S3a – ROC for HRS and HRS + Area Deprivation Index**

**
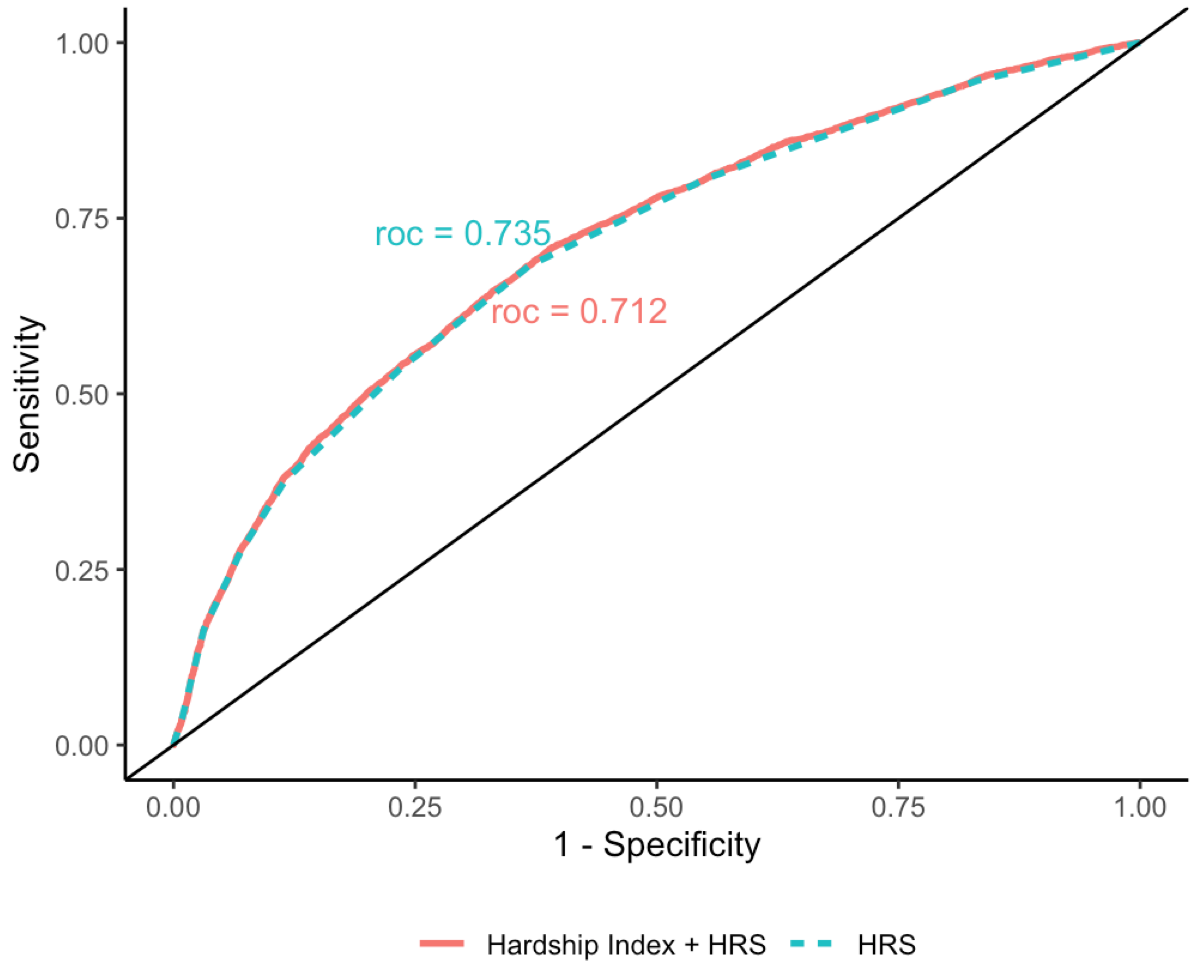
Figure S3b – ROC for HRS and HRS + Hardship Index**

**Supplemental Tables**

Table S1: PCA Component Scores, all patients

|  | **Low Income** | **No High School Diploma** | **Age 65 and above or Disabled** | **No Vehicle and Multiunit Living** |
| --- | --- | --- | --- | --- |
| **Poverty** | 0.793 |  |  |  |
| **Unemployment** | 0.860 |  |  |  |
| **Per Capita Income** | -0.796 |  |  |  |
| **Single Parent Household** | 0.822 |  |  |  |
| **Minority** | 0.792 |  |  |  |
| **Violent Crime** | 0.793 |  |  |  |
| **No High School Diploma** |  | 0.785 |  |  |
| **Limited English** |  | 0.870 |  |  |
| **Crowded Households** |  | 0.680 |  |  |
| **Disabled** |  |  | 0.805 |  |
| **Age 65 and Above** |  |  | 0.875 |  |
| **No Vehicle Households** |  |  |  | 0.822 |
| **Multiunit Living** |  |  |  | 0.842 |

Table S2: PCA Component Scores, randomly-sampled balanced dataset

|  | **Low Income** | **No High School Diploma** | **Age 65 and above or Disabled** | **No Vehicle and Multiunit Living** |
| --- | --- | --- | --- | --- |
| **Poverty** | 0.802 |  |  |  |
| **Unemployment** | 0.876 |  |  |  |
| **Per Capita Income** | -0.715 |  |  |  |
| **Single Parent Household** | 0.858 |  |  |  |
| **Minority** | 0.742 |  |  |  |
| **Violent Crime** | 0.825 |  |  |  |
| **No High School Diploma** |  | 0.842 |  |  |
| **Limited English** |  | 0.895 |  |  |
| **Crowded Households** |  | 0.8 |  |  |
| **Disabled** |  |  | 0.809 |  |
| **Age 65 and Above** |  |  | 0.896 |  |
| **No Vehicle Households** |  |  |  | 0.827 |
| **Multiunit Living** |  |  |  | 0.893 |

Table S3: PCA Component Scores, patients with heart failure

|  | **Low Income** | **No High School Diploma** | **Age 65 and above or Disabled** | **No Vehicle and Multiunit Living** |
| --- | --- | --- | --- | --- |
| **Poverty** | 0.793 |  |  |  |
| **Unemployment** | 0.860 |  |  |  |
| **Per Capita Income** | -0.796 |  |  |  |
| **Single Parent Household** | 0.822 |  |  |  |
| **Minority** | 0.792 |  |  |  |
| 0.817**Violent Crime** | 0.793 |  |  |  |
| **No High School Diploma** |  | 0.785 |  |  |
| **Limited English** |  | 0.870 |  |  |
| **Crowded Households** |  | 0.680 |  |  |
| **Disabled** |  |  | 0.805 |  |
| **Age 65 and Above** |  |  | 0.875 |  |
| **No Vehicle Households** |  |  |  | 0.822 |
| **Multiunit Living** |  |  |  | 0.842 |

|  | **Low Income** | **No High School Diploma** | **Age 65 and above or Disabled** | **No Vehicle and Multiunit Living** |
| --- | --- | --- | --- | --- |
| **Poverty** | 0.716 |  |  |  |
| **Unemployment** | 0.826 |  |  |  |
| **Per Capita Income** | -0.833 |  |  |  |
| **Single Parent Household** | 0.753 |  |  |  |
| **Minority** | 0.794 |  |  |  |
| **Violent Crime** | 0.766 |  |  |  |
| **No High School Diploma** |  | 0.741 |  |  |
| **Limited English** |  | 0.891 |  |  |
| **Disabled** |  |  | 0.854 |  |
| **Age 65 and Above** |  |  | 0.865 |  |
| **No Vehicle Households** |  |  |  | 0.877 |
| **Multiunit Living** |  |  |  | 0.802 |

Table S4: PCA Component Scores, patients with atrial fibrillation

|  | **Low Income** | **No High School Diploma** | **Age 65 and above or Disabled** | **No Vehicle and Multiunit Living** |
| --- | --- | --- | --- | --- |
| **Poverty** | 0.810 |  |  |  |
| **Unemployment** | 0.860 |  |  |  |
| **Per Capita Income** | -0.815 |  |  |  |
| **Single Parent Household** | 0.820 |  |  |  |
| **Minority** | 0.789 |  |  |  |
| **Violent Crime** | 0.837 |  |  |  |
| **No High School Diploma** |  | 0.753 |  |  |
| **Limited English** |  | 0.891 |  |  |
| **Crowded Households** |  | 0.690 |  |  |
| **Disabled** |  |  | 0.760 |  |
| **Age 65 and Above** |  |  | 0.866 |  |
| **No Vehicle Households** |  |  |  | 0.820 |
| **Multiunit Living** |  |  |  | 0.818 |

Table S5: PCA Component Scores, patients with coronary artery disease

|  | **Low Income** | **No High School Diploma** | **Age 65 and above or Disabled** | **No Vehicle and Multiunit Living** |
| --- | --- | --- | --- | --- |
| **Poverty** | 0.747 |  |  |  |
| **Unemployment** | 0.834 |  |  |  |
| **Per Capita Income** | -0.836 |  |  |  |
| **Single Parent Household** | 0.814 |  |  |  |
| **Minority** | 0.828 |  |  |  |
| **Violent Crime** | 0.800 |  |  |  |
| **No High School Diploma** |  | 0.709 |  |  |
| **Limited English** |  | 0.892 |  |  |
| **Disabled** |  |  | 0.751 |  |
| **Age 65 and Above** |  |  | 0.871 |  |
| **No Vehicle Households** |  |  |  | 0.896 |
| **Multiunit Living** |  |  |  | 0.739 |

Table S6: PCA Component Scores, patients with COPD

|  | **Low Income** | **Age 65 and above or Disabled** | | **No Vehicle and Multiunit Living** | |
| --- | --- | --- | --- | --- | --- |
| **Unemployment** | 0.75 |  |  | |  |
| **Per Capita Income** | -0.866 |  |  | |  |
| **Minority** | 0.825 |  |  | |  |
| **Violent Crime** | 0.685 |  |  | |  |
| **No High School Diploma** | 0.716 |  |  | |  |
| **Disabled** |  | 0.700 |  | |  |
| **Age 65 and Above** |  | 0.905 |  | |  |
| **No Vehicle Households** |  |  | 0.914 | |  |
| **Multiunit Living** |  |  | 0.654 | |  |

Table S7: PCA Component Scores, patients with liver disease

|  | | **Low Income** | | **No High School Diploma** | | **Age 65 and above or Disabled** | | **No Vehicle and Multiunit Living** | |
| --- | --- | --- | --- | --- | --- | --- | --- | --- | --- |
| **Poverty** | | 0.793 | |  | |  | |  | |
| **Unemployment** | | 0.860 | |  | |  | |  | |
| **Per Capita Income** | | -0.796 | |  | |  | |  | |
| **Single Parent Household** | | 0.822 | |  | |  | |  | |
| **Minority** | | 0.792 | |  | |  | |  | |
| 0.817**Violent Crime** | | 0.793 | |  | |  | |  | |
| **No High School Diploma** | |  | | 0.785 | |  | |  | |
| **Limited English** | |  | | 0.870 | |  | |  | |
| **Crowded Households** | |  | | 0.680 | |  | |  | |
| **Disabled** | |  | |  | | 0.805 | |  | |
| **Age 65 and Above** | |  | |  | | 0.875 | |  | |
| **No Vehicle Households** | |  | |  | |  | | 0.822 | |
| **Multiunit Living** | |  | |  | |  | | 0.842 | |
|  | **Low Income** | | **No High School Diploma** | | **Age 65 and above or Disabled** | | **No Vehicle and Multiunit Living** | |  |
| **Poverty** | 0.851 | |  | |  | |  | |  |
| **Unemployment** | 0.836 | |  | |  | |  | |  |
| **Per Capita Income** | -0.739 | |  | |  | |  | |  |
| **Single Parent Household** | 0.830 | |  | |  | |  | |  |
| **Minority** | 0.740 | |  | |  | |  | |  |
| **Violent Crime** | 0.832 | |  | |  | |  | |  |
| **No High School Diploma** |  | | 0.937 | |  | |  | |  |
| **Limited English** |  | | 0.879 | |  | |  | |  |
| **Disabled** |  | |  | | 0.839 | |  | |  |
| **Age 65 and Above** |  | |  | | 0.840 | |  | |  |
| **No Vehicle Households** |  | |  | |  | | 0.665 | |  |
| **Multiunit Living** |  | |  | |  | | 0.915 | |  |

Table S8: PCA Component Scores, patients with obesity

|  | **Low Income** | **No High School Diploma** | **Age 65 and above or Disabled** | **No Vehicle and Multiunit Living** | |
| --- | --- | --- | --- | --- | --- |
| **Poverty** | 0.824 |  |  |  |  |
| **Unemployment** | 0.895 |  |  |  |  |
| **Per Capita Income** | -0.748 |  |  |  |  |
| **Single Parent Household** | 0.867 |  |  |  |  |
| **Minority** | 0.781 |  |  |  |  |
| **Violent Crime** | 0.822 |  |  |  |  |
| **No High School Diploma** |  | 0.760 |  |  |  |
| **Limited English** |  | 0.892 |  |  |  |
| **Crowded Households** |  | 0.697 |  |  |  |
| **Disabled** |  |  | 0.781 |  |  |
| **Age 65 and Above** |  |  | 0.888 |  |  |
| **No Vehicle Households** |  |  |  | 0.825 |  |
| **Multiunit Living** |  |  |  | 0.858 |  |

Table S9: PCA Component Scores, patients with pulmonary disease

|  | **Low Income** | **No High School Diploma** | **Age 65 and above or Disabled** | **No Vehicle and Multiunit Living** | |
| --- | --- | --- | --- | --- | --- |
| **Poverty** | 0.732 |  |  |  |  |
| **Unemployment** | 0.815 |  |  |  |  |
| **Per Capita Income** | -0.847 |  |  |  |  |
| **Single Parent Household** | 0.737 |  |  |  |  |
| **Minority** | 0.783 |  |  |  |  |
| **Violent Crime** | 0.761 |  |  |  |  |
| **No High School Diploma** |  | 0.632 |  |  |  |
| **Limited English** |  | 0.868 |  |  |  |
| **Disabled** |  |  | 0.864 |  |  |
| **Age 65 and Above** |  |  | 0.849 |  |  |
| **No Vehicle Households** |  |  |  | 0.849 |  |
| **Multiunit Living** |  |  |  | 0.801 |  |

Table S10: PCA Component Scores, patients with valvular heart disease

|  | **Low Income** | **No High School Diploma** | **Age 65 and above or Disabled** | **No Vehicle and Multiunit Living** | |
| --- | --- | --- | --- | --- | --- |
| **Poverty** | 0.787 |  |  |  |  |
| **Unemployment** | 0.887 |  |  |  |  |
| **Per Capita Income** | -0.758 |  |  |  |  |
| **Single Parent Household** | 0.872 |  |  |  |  |
| **Minority** | 0.803 |  |  |  |  |
| **Violent Crime** | 0.828 |  |  |  |  |
| **No High School Diploma** |  | 0.860 |  |  |  |
| **Limited English** |  | 0.910 |  |  |  |
| **Crowded Households** |  | 0.753 |  |  |  |
| **Disabled** |  |  | 0.707 |  |  |
| **Age 65 and Above** |  |  | 0.890 |  |  |
| **No Vehicle Households** |  |  |  | 0.851 |  |
| **Multiunit Living** |  |  |  | 0.870 |  |

Table S11: PCA Component Scores, female patients

|  | **Low Income** | **No High School Diploma** | **Age 65 and above or Disabled** | **No Vehicle and Multiunit Living** | |
| --- | --- | --- | --- | --- | --- |
| **Poverty** | 0.770 |  |  |  |  |
| **Unemployment** | 0.858 |  |  |  |  |
| **Per Capita Income** | -0.811 |  |  |  |  |
| **Single Parent Household** | 0.809 |  |  |  |  |
| **Minority** | 0.789 |  |  |  |  |
| **Violent Crime** | 0.810 |  |  |  |  |
| **No High School Diploma** |  | 0.782 |  |  |  |
| **Limited English** |  | 0.873 |  |  |  |
| **Crowded Households** |  | 0.658 |  |  |  |
| **Disabled** |  |  | 0.818 |  |  |
| **Age 65 and Above** |  |  | 0.864 |  |  |
| **No Vehicle Households** |  |  |  | 0.833 |  |
| **Multiunit Living** |  |  |  | 0.833 |  |

Table S12: PCA Component Scores, male patients

|  | **Low Income** | **No High School Diploma** | **Age 65 and above or Disabled** | **No Vehicle and Multiunit Living** | |
| --- | --- | --- | --- | --- | --- |
| **Poverty** | 0.818 |  |  |  |  |
| **Unemployment** | 0.861 |  |  |  |  |
| **Per Capita Income** | -0.775 |  |  |  |  |
| **Single Parent Household** | 0.835 |  |  |  |  |
| **Minority** | 0.801 |  |  |  |  |
| **Violent Crime** | 0.827 |  |  |  |  |
| **No High School Diploma** |  | 0.788 |  |  |  |
| **Limited English** |  | 0.869 |  |  |  |
| **Crowded Households** |  | 0.713 |  |  |  |
| **Disabled** |  |  | 0.777 |  |  |
| **Age 65 and Above** |  |  | 0.895 |  |  |
| **No Vehicle Households** |  |  |  | 0.813 |  |
| **Multiunit Living** |  |  |  | 0.847 |  |

Table S13: Linear Regression Estimates, all patients

|  | **Estimate** | **Standard Error** | **p value** |  |
| --- | --- | --- | --- | --- |
| **Low Income** | -0.008 | 0.013 | 0.545 |  |
| **No High School** | 0.062 | 0.013 | 0.000 | *** |
| **Aged and Disabled** | -0.017 | 0.013 | 0.185 |  |
| **No Vehicle and Multiunit Living** | -0.060 | 0.013 | 0.000 | *** |
| **Congestive Heart Failure** | 0.142 | 0.037 | 0.000 | *** |
| **Valvular Disease** | 0.480 | 0.048 | 0.000 | *** |
| **Hypertension** | -0.023 | 0.030 | 0.437 |  |
| **Diabetes Mellitus** | 0.093 | 0.031 | 0.003 | ** |
| **Renal Disease** | 0.740 | 0.033 | 0.000 | *** |
| **Liver Disease** | 0.688 | 0.047 | 0.000 | *** |
| **Chronic Obstructive Pulmonary Disease** | 0.345 | 0.036 | 0.000 | *** |
| **Atrial Fibrillation** | 0.169 | 0.043 | 0.000 | *** |
| **Dyslipidemia** | -0.278 | 0.033 | 0.000 | *** |
| **Coronary Artery Disease** | -0.002 | 0.036 | 0.950 |  |
|  |  |  |  |  |
| **R-squared** | 0.05581 |  |  |  |
| **Adjusted R-squared** | 0.05532 |  |  |  |

Table S14: Linear Regression Estimates, randomly-sampled balanced dataset

|  | **Estimate** | **Standard Error** | **p value** |  |
| --- | --- | --- | --- | --- |
| **Low Income** | -0.038 | 0.022 | 0.080 |  |
| **No High School** | 0.045 | 0.021 | 0.038 | * |
| **Aged and Disabled** | -0.043 | 0.021 | 0.043 | * |
| **No Vehicle and Multiunit Living** | -0.083 | 0.021 | 0.000 | *** |
| **Congestive Heart Failure** | 0.042 | 0.062 | 0.504 |  |
| **Valvular Disease** | 0.481 | 0.078 | 0.000 | *** |
| **Hypertension** | -0.070 | 0.049 | 0.152 |  |
| **Diabetes Mellitus** | 0.193 | 0.052 | 0.000 | ** |
| **Renal Disease** | 0.836 | 0.056 | 0.000 | *** |
| **Liver Disease** | 0.762 | 0.074 | 0.000 | *** |
| **Chronic Obstructive Pulmonary Disease** | 0.399 | 0.062 | 0.000 | *** |
| **Atrial Fibrillation** | 0.112 | 0.071 | 0.114 |  |
| **Dyslipidemia** | -0.375 | 0.052 | 0.000 | *** |
| **Coronary Artery Disease** | 0.159 | 0.059 | 0.007 | ** |
|  |  |  |  |  |
| **R-squared** | 0.06422 |  |  |  |
| **Adjusted R-squared** | 0.06296 |  |  |  |

Table S15: Linear Regression Estimates, patients with heart failure

|  | **Estimate** | **Standard Error** | **p value** |  |
| --- | --- | --- | --- | --- |
| **Low Income** | -0.044 | 0.053 | 0.413 |  |
| **No High School** | -0.138 | 0.054 | 0.010 | * |
| **Aged and Disabled** | 0.018 | 0.053 | 0.743 |  |
| **No Vehicle and Multiunit Living** | -0.208 | 0.053 | 0.000 | *** |
|  |  |  |  |  |
| **R-squared** | 0.01426 |  |  |  |
| **Adjusted R-squared** | 0.000152 |  |  |  |
|  |  |  |  |  |
|  |  |  |  |  |
|  |  |  |  |  |
|  |  |  |  |  |
|  |  |  |  |  |
|  |  |  |  |  |
|  |  |  |  |  |
|  |  |  |  |  |
|  |  |  |  |  |
|  |  |  |  |  |

Table S16: Linear Regression Estimates, patients with atrial fibrillation

|  | **Estimate** | **Standard Error** | **p value** |  |
| --- | --- | --- | --- | --- |
| **Low Income** | 0.145 | 0.085 | 0.089 |  |
| **No High School** | -0.008 | 0.084 | 0.920 |  |
| **Aged and Disabled** | 0.108 | 0.085 | 0.206 |  |
| **No Vehicle and Multiunit Living** | -0.018 | 0.085 | 0.830 |  |
|  |  |  |  |  |
| **R-squared** | 0.01056 |  |  |  |
| **Adjusted R-squared** | 0.001027 |  |  |  |
|  |  |  |  |  |
|  |  |  |  |  |
|  |  |  |  |  |
|  |  |  |  |  |
|  |  |  |  |  |
|  |  |  |  |  |
|  |  |  |  |  |
|  |  |  |  |  |
|  |  |  |  |  |
|  |  |  |  |  |

Table S17: Linear Regression Estimates, patients with coronary artery disease

|  | **Estimate** | **Standard Error** | **p value** |  |
| --- | --- | --- | --- | --- |
| **Low Income** | 0.022 | 0.076 | 0.769 |  |
| **No High School** | 0.079 | 0.076 | 0.299 |  |
| **Aged and Disabled** | 0.295 | 0.076 | 0.000 | *** |
| **No Vehicle and Multiunit Living** | 0.148 | 0.077 | 0.050 |  |
|  |  |  |  |  |
| **R-squared** | 0.04822 |  |  |  |
| **Adjusted R-squared** | 0.038410 |  |  |  |
|  |  |  |  |  |
|  |  |  |  |  |
|  |  |  |  |  |
|  |  |  |  |  |
|  |  |  |  |  |
|  |  |  |  |  |
|  |  |  |  |  |
|  |  |  |  |  |
|  |  |  |  |  |
|  |  |  |  |  |

Table S18: Linear Regression Estimates, patients with COPD

|  | **Estimate** | **Standard Error** | **p value** |  |
| --- | --- | --- | --- | --- |
| **Low Income** | 0.129 | 0.071 | 0.069 |  |
| **Aged and Disabled** | 0.152 | 0.071 | 0.033 |  |
| **No Vehicle and Multiunit Living** | -0.115 | 0.071 | 0.107 |  |
|  |  |  |  |  |
| **R-squared** | 0.0151 |  |  |  |
| **Adjusted R-squared** | 0.010800 |  |  |  |
|  |  |  |  |  |
|  |  |  |  |  |
|  |  |  |  |  |
|  |  |  |  |  |
|  |  |  |  |  |
|  |  |  |  |  |
|  |  |  |  |  |
|  |  |  |  |  |
|  |  |  |  |  |
|  |  |  |  |  |

Table S19: Linear Regression Estimates, patients with liver disease

|  | **Estimate** | **Standard Error** | **p value** |  |
| --- | --- | --- | --- | --- |
| **Low Income** | -0.063 | 0.162 | 0.698 |  |
| **No High School** | -0.103 | 0.164 | 0.532 |  |
| **Aged and Disabled** | 0.319 | 0.162 | 0.051 |  |
| **No Vehicle and Multiunit Living** | 0.036 | 0.162 | 0.824 |  |
|  |  |  |  |  |
| **R-squared** | 0.03136 |  |  |  |
| **Adjusted R-squared** | 0.003077 |  |  |  |
|  |  |  |  |  |
|  |  |  |  |  |
|  |  |  |  |  |
|  |  |  |  |  |
|  |  |  |  |  |
|  |  |  |  |  |
|  |  |  |  |  |
|  |  |  |  |  |
|  |  |  |  |  |
|  |  |  |  |  |

Table S20: Linear Regression Estimates, patients with obesity

|  | **Estimate** | **Standard Error** | **p value** |  |
| --- | --- | --- | --- | --- |
| **Low Income** | 0.022 | 0.076 | 0.769 |  |
| **No High School** | 0.079 | 0.076 | 0.299 |  |
| **Aged and Disabled** | 0.295 | 0.076 | 0.000 | *** |
| **No Vehicle and Multiunit Living** | 0.148 | 0.077 | 0.050 |  |
|  |  |  |  |  |
| **R-squared** | 0.04822 |  |  |  |
| **Adjusted R-squared** | 0.038410 |  |  |  |
|  |  |  |  |  |
|  |  |  |  |  |
|  |  |  |  |  |
|  |  |  |  |  |
|  |  |  |  |  |
|  |  |  |  |  |
|  |  |  |  |  |
|  |  |  |  |  |
|  |  |  |  |  |
|  |  |  |  |  |

|  | **Estimate** | **Standard Error** | **p value** |  |
| --- | --- | --- | --- | --- |
| **Low Income** | 0.127 | 0.050 | 0.010 | * |
| **No High School** | -0.041 | 0.050 | 0.404 |  |
| **Aged and Disabled** | -0.055 | 0.050 | 0.274 |  |
| **No Vehicle and Multiunit Living** | -0.005 | 0.050 | 0.924 |  |
|  |  |  |  |  |
| **R-squared** | 0.02947 |  |  |  |
| **Adjusted R-squared** | 0.015710 |  |  |  |
|  |  |  |  |  |
|  |  |  |  |  |
|  |  |  |  |  |
|  |  |  |  |  |
|  |  |  |  |  |
|  |  |  |  |  |
|  |  |  |  |  |
|  |  |  |  |  |
|  |  |  |  |  |
|  |  |  |  |  |

Table S21: Linear Regression Estimates, patients with pulmonary disease

|  | **Estimate** | **Standard Error** | **p value** |  |
| --- | --- | --- | --- | --- |
| **Low Income** | 0.016 | 0.050 | 0.764 |  |
| **No High School** | 0.087 | 0.050 | 0.001 | *** |
| **Aged and Disabled** | 0.179 | 0.050 | 0.410 |  |
| **No Vehicle and Multiunit Living** | -0.044 | 0.050 | 0.105 |  |
|  |  |  |  |  |
| **R-squared** | 0.01273 |  |  |  |
| **Adjusted R-squared** | 0.009210 |  |  |  |
|  |  |  |  |  |
|  |  |  |  |  |
|  |  |  |  |  |
|  |  |  |  |  |
|  |  |  |  |  |
|  |  |  |  |  |
|  |  |  |  |  |
|  |  |  |  |  |
|  |  |  |  |  |
|  |  |  |  |  |

Table S22: Linear Regression Estimates, patients with valvular heart disease

|  | **Estimate** | **Standard Error** | **p value** |  |
| --- | --- | --- | --- | --- |
| **Low Income** | 0.402 | 0.125 | 0.002 | ** |
| **No High School** | -0.053 | 0.125 | 0.670 |  |
| **Aged and Disabled** | -0.164 | 0.125 | 0.193 |  |
| **No Vehicle and Multiunit Living** | -0.078 | 0.125 | 0.535 |  |
|  |  |  |  |  |
| **R-squared** | 0.09616 |  |  |  |
| **Adjusted R-squared** | 0.065780 |  |  |  |
|  |  |  |  |  |
|  |  |  |  |  |
|  |  |  |  |  |
|  |  |  |  |  |
|  |  |  |  |  |
|  |  |  |  |  |
|  |  |  |  |  |
|  |  |  |  |  |
|  |  |  |  |  |
|  |  |  |  |  |

Table S23: Linear Regression Estimates, female patients

|  | **Estimate** | **Standard Error** | **p value** |  |
| --- | --- | --- | --- | --- |
| **Low Income** | 0.037 | 0.017 | 0.026 | * |
| **No High School** | 0.022 | 0.017 | 0.185 |  |
| **Aged and Disabled** | -0.017 | 0.016 | 0.305 |  |
| **No Vehicle and Multiunit Living** | -0.033 | 0.016 | 0.039 | * |
| **Congestive Heart Failure** | 0.136 | 0.049 | 0.005 | ** |
| **Valvular Disease** | 0.493 | 0.061 | 0.000 | *** |
| **Hypertension** | -0.061 | 0.038 | 0.106 |  |
| **Diabetes Mellitus** | 0.045 | 0.040 | 0.259 |  |
| **Renal Disease** | 0.819 | 0.044 | 0.000 | *** |
| **Liver Disease** | 0.619 | 0.063 | 0.000 | *** |
| **Chronic Obstructive Pulmonary Disease** | 0.251 | 0.046 | 0.000 | *** |
| **Atrial Fibrillation** | -0.004 | 0.060 | 0.942 |  |
| **Dyslipidemia** | -0.262 | 0.042 | 0.000 | *** |
| **Coronary Artery Disease** | 0.054 | 0.048 | 0.260 |  |
|  |  |  |  |  |
| **R-squared** | 0.05061 |  |  |  |
| **Adjusted R-squared** | 0.04979 |  |  |  |

Table S24: Linear Regression Estimates, male patients

|  | **Estimate** | **Standard Error** | **p value** |  |
| --- | --- | --- | --- | --- |
| **Low Income** | -0.075 | 0.022 | 0.001 | *** |
| **No High School** | 0.106 | 0.021 | 0.000 | *** |
| **Aged and Disabled** | -0.013 | 0.021 | 0.550 |  |
| **No Vehicle and Multiunit Living** | -0.096 | 0.021 | 0.000 | *** |
| **Congestive Heart Failure** | 0.161 | 0.058 | 0.006 | ** |
| **Valvular Disease** | 0.472 | 0.076 | 0.000 | *** |
| **Hypertension** | 0.027 | 0.048 | 0.573 |  |
| **Diabetes Mellitus** | 0.160 | 0.051 | 0.002 | ** |
| **Renal Disease** | 0.661 | 0.051 | 0.000 | *** |
| **Liver Disease** | 0.761 | 0.069 | 0.000 | *** |
| **Chronic Obstructive Pulmonary Disease** | 0.492 | 0.058 | 0.000 | *** |
| **Atrial Fibrillation** | 0.333 | 0.063 | 0.000 | *** |
| **Dyslipidemia** | -0.300 | 0.052 | 0.000 | *** |
| **Coronary Artery Disease** | -0.067 | 0.055 | 0.000 | *** |
|  |  |  |  |  |
| **R-squared** | 0.06769 |  |  |  |
| **Adjusted R-squared** | 0.06650 |  |  |  |

Table S25: PCA Component Scores, all patients

|  | **Low Income** | **No High School Diploma** |  |  |
| --- | --- | --- | --- | --- |
| **Poverty (%)** | 0.787 |  |  |  |
| **Aged 16+, Unemployment (%)** | 0.941 |  |  |  |
| **Aged 18 and Under, Age 64 and Above (%)** | 0.81 |  |  |  |
| **Per Capita Income ($)** | -0.812 |  |  |  |
| **Crowded Households (%)** |  | 0.974 |  |  |
| **Age 25+, No High School Diploma** |  | 0.885 |  |  |
|  |  |  |  |  |
|  |  |  |  |  |
|  |  |  |  |  |
|  |  |  |  |  |
|  |  |  |  |  |
|  |  |  |  |  |
|  |  |  |  |  |

Table S26: PCA Component Scores, randomly-sampled balanced dataset

|  | **Low Income** | **No High School Diploma** |  |  |
| --- | --- | --- | --- | --- |
| **Poverty (%)** | 0.734 |  |  |  |
| **Aged 16+, Unemployment (%)** | 0.950 |  |  |  |
| **Aged 18 and Under, Age 64 and Above (%)** | 0.770 |  |  |  |
| **Per Capita Income ($)** | -0.777 |  |  |  |
| **Crowded Households (%)** |  | 0.975 |  |  |
| **Age 25+, No High School Diploma** |  | 0.929 |  |  |
|  |  |  |  |  |
|  |  |  |  |  |
|  |  |  |  |  |
|  |  |  |  |  |
|  |  |  |  |  |
|  |  |  |  |  |
|  |  |  |  |  |

Table S27: PCA Component Scores, patients with heart failure

|  | **Low Income** | **No High School Diploma** |  |  |
| --- | --- | --- | --- | --- |
| **Poverty (%)** | 0.759 |  |  |  |
| **Aged 16+, Unemployment (%)** | 0.926 |  |  |  |
| **Aged 18 and Under, Age 64 and Above (%)** | 0.790 |  |  |  |
| **Per Capita Income ($)** | -0.802 |  |  |  |
| **Crowded Households (%)** |  | 0.969 |  |  |
| **Age 25+, No High School Diploma** |  | 0.867 |  |  |
|  |  |  |  |  |
|  |  |  |  |  |
|  |  |  |  |  |
|  |  |  |  |  |
|  |  |  |  |  |
|  |  |  |  |  |
|  |  |  |  |  |

Table S28: PCA Component Scores, patients with atrial fibrillation

|  | **Low Income** | **No High School Diploma** |  |  |
| --- | --- | --- | --- | --- |
| **Poverty (%)** | 0.778 |  |  |  |
| **Aged 16+, Unemployment (%)** | 0.953 |  |  |  |
| **Aged 18 and Under, Age 64 and Above (%)** | 0.831 |  |  |  |
| **Per Capita Income ($)** | -0.819 |  |  |  |
| **Crowded Households (%)** |  | 0.98 |  |  |
| **Age 25+, No High School Diploma** |  | 0.881 |  |  |
|  |  |  |  |  |

Table S29: PCA Component Scores, patients with coronary artery disease

|  | **Low Income** | **No High School Diploma** |  |  |
| --- | --- | --- | --- | --- |
| **Poverty (%)** | 0.799 |  |  |  |
| **Aged 16+, Unemployment (%)** | 0.941 |  |  |  |
| **Aged 18 and Under, Age 64 and Above (%)** | 0.792 |  |  |  |
| **Per Capita Income ($)** | -0.814 |  |  |  |
| **Crowded Households (%)** |  | 0.974 |  |  |
| **Age 25+, No High School Diploma** |  | 0.832 |  |  |
|  |  |  |  |  |

Table S30: PCA Component Scores, patients with COPD

|  | **Low Income** | **No High School Diploma** |  |  |
| --- | --- | --- | --- | --- |
| **Poverty (%)** | 0.782 |  |  |  |
| **Aged 16+, Unemployment (%)** | 0.939 |  |  |  |
| **Aged 18 and Under, Age 64 and Above (%)** | 0.808 |  |  |  |
| **Per Capita Income ($)** | -0.836 |  |  |  |
| **Crowded Households (%)** |  | 0.964 |  |  |
| **Age 25+, No High School Diploma** |  | 0.821 |  |  |
|  |  |  |  |  |

Table S31: PCA Component Scores, patients with liver diseases

|  | **Low Income** | **No High School Diploma** |  |  |
| --- | --- | --- | --- | --- |
| **Poverty (%)** | 0.744 |  |  |  |
| **Aged 16+, Unemployment (%)** | 0.925 |  |  |  |
| **Aged 18 and Under, Age 64 and Above (%)** | 0.636 |  |  |  |
| **Per Capita Income ($)** | -0.758 |  |  |  |
| **Crowded Households (%)** |  | 0.959 |  |  |
| **Age 25+, No High School Diploma** |  | 0.952 |  |  |
|  |  |  |  |  |

Table S32: PCA Component Scores, patients with obesity

|  | **Low Income** | **No High School Diploma** |  |  |
| --- | --- | --- | --- | --- |
| **Poverty (%)** | 0.708 |  |  |  |
| **Aged 16+, Unemployment (%)** | 0.939 |  |  |  |
| **Aged 18 and Under, Age 64 and Above (%)** | 0.825 |  |  |  |
| **Per Capita Income ($)** | -0.804 |  |  |  |
| **Crowded Households (%)** |  | 0.976 |  |  |
| **Age 25+, No High School Diploma** |  | 0.891 |  |  |
|  |  |  |  |  |

Table S33: PCA Component Scores, patients with pulmonary disease

|  | **Low Income** | **No High School Diploma** |  |  |
| --- | --- | --- | --- | --- |
| **Poverty (%)** | 0.762 |  |  |  |
| **Aged 16+, Unemployment (%)** | 0.932 |  |  |  |
| **Aged 18 and Under, Age 64 and Above (%)** | 0.802 |  |  |  |
| **Per Capita Income ($)** | -0.841 |  |  |  |
| **Crowded Households (%)** |  | 0.968 |  |  |
| **Age 25+, No High School Diploma** |  | 0.834 |  |  |
|  |  |  |  |  |

Table S34: PCA Component Scores, patients with valvular heart disease

|  | **Low Income** | **No High School Diploma** |  |  |
| --- | --- | --- | --- | --- |
| **Poverty (%)** | 0.829 |  |  |  |
| **Aged 16+, Unemployment (%)** | 0.951 |  |  |  |
| **Aged 18 and Under, Age 64 and Above (%)** | 0.809 |  |  |  |
| **Per Capita Income ($)** | -0.773 |  |  |  |
| **Crowded Households (%)** |  | 0.971 |  |  |
| **Age 25+, No High School Diploma** |  | 0.921 |  |  |
|  |  |  |  |  |

Table S35: PCA Component Scores, female patients

|  | **Low Income** | **No High School Diploma** |  |  |
| --- | --- | --- | --- | --- |
| **Poverty (%)** | 0.784 |  |  |  |
| **Aged 16+, Unemployment (%)** | 0.938 |  |  |  |
| **Aged 18 and Under, Age 64 and Above (%)** | 0.807 |  |  |  |
| **Per Capita Income ($)** | -0.813 |  |  |  |
| **Crowded Households (%)** |  | 0.974 |  |  |
| **Age 25+, No High School Diploma** |  | 0.89 |  |  |
|  |  |  |  |  |

Table S36: PCA Component Scores, male patients

|  | **Low Income** | **No High School Diploma** |  |  |
| --- | --- | --- | --- | --- |
| **Poverty (%)** | 0.788 |  |  |  |
| **Aged 16+, Unemployment (%)** | 0.946 |  |  |  |
| **Aged 18 and Under, Age 64 and Above (%)** | 0.810 |  |  |  |
| **Per Capita Income ($)** | -0.808 |  |  |  |
| **Crowded Households (%)** |  | 0.974 |  |  |
| **Age 25+, No High School Diploma** |  | 0.879 |  |  |
|  |  |  |  |  |

Table S37: Linear Regression Estimates, all patients

|  | **Estimate** | **Standard Error** | **p value** |  |
| --- | --- | --- | --- | --- |
| **Low Income** | -0.009 | 0.013 | 0.516 |  |
| **No High School** | 0.069 | 0.013 | 0.000 | *** |
| **Congestive Heart Failure** | 0.139 | 0.037 | 0.000 | *** |
| **Valvular Disease** | 0.481 | 0.048 | 0.000 | *** |
| **Hypertension** | -0.024 | 0.030 | 0.418 |  |
| **Diabetes Mellitus** | 0.096 | 0.031 | 0.002 | ** |
| **Renal Disease** | 0.740 | 0.033 | 0.000 | *** |
| **Liver Disease** | 0.697 | 0.046 | 0.000 | *** |
| **Chronic Obstructive Pulmonary Disease** | 0.339 | 0.036 | 0.000 | *** |
| **Atrial Fibrillation** | 0.163 | 0.043 | 0.000 | *** |
| **Dyslipidemia** | -0.277 | 0.032 | 0.000 | *** |
| **Coronary Artery Disease** | -0.003 | 0.036 | 0.923 |  |
|  |  |  |  |  |
| **R-squared** | 0.05521 |  |  |  |
| **Adjusted R-squared** | 0.05479 |  |  |  |

Table S38: PCA Component Scores, randomly-sampled balanced dataset

|  | **Estimate** | **Standard Error** | **p value** |  |
| --- | --- | --- | --- | --- |
| **Low Income** | -0.043 | 0.022 | 0.049 | * |
| **No High School** | 0.059 | 0.021 | 0.006 | ** |
| **Congestive Heart Failure** | 0.041 | 0.062 | 0.506 |  |
| **Valvular Disease** | 0.482 | 0.078 | 0.000 | *** |
| **Hypertension** | -0.076 | 0.049 | 0.117 |  |
| **Diabetes Mellitus** | 0.196 | 0.052 | 0.000 | *** |
| **Renal Disease** | 0.833 | 0.056 | 0.000 | *** |
| **Liver Disease** | 0.778 | 0.074 | 0.000 | *** |
| **Chronic Obstructive Pulmonary Disease** | 0.389 | 0.062 | 0.000 | *** |
| **Atrial Fibrillation** | 0.103 | 0.071 | 0.149 |  |
| **Dyslipidemia** | -0.375 | 0.052 | 0.000 | *** |
| **Coronary Artery Disease** | 0.153 | 0.059 | 0.010 | *** |
|  |  |  |  |  |
| **R-squared** | 0.06286 |  |  |  |
| **Adjusted R-squared** | 0.06178 |  |  |  |

Table S39: PCA Component Scores, patients with heart failure

|  | **Estimate** | **Standard Error** | **p value** |  |
| --- | --- | --- | --- | --- |
| **Low Income** | 0.060 | 0.054 | 0.261 |  |
| **No High School** | -0.113 | 0.055 | 0.040 | * |
|  |  |  |  |  |
| **R-squared** | 0.003444 |  |  |  |
| **Adjusted R-squared** | 0.002179 |  |  |  |
|  |  |  |  |  |
|  |  |  |  |  |
|  |  |  |  |  |
|  |  |  |  |  |
|  |  |  |  |  |
|  |  |  |  |  |
|  |  |  |  |  |
|  |  |  |  |  |
|  |  |  |  |  |
|  |  |  |  |  |

Table S40: PCA Component Scores, patients with atrial fibrillation

|  | **Estimate** | **Standard Error** | **p value** |  |
| --- | --- | --- | --- | --- |
| **Low Income** | 0.130 | 0.087 | 0.134 |  |
| **No High School** | 0.048 | 0.084 | 0.566 |  |
|  |  |  |  |  |
| **R-squared** | 0.006093 |  |  |  |
| **Adjusted R-squared** | 0.001326 |  |  |  |
|  |  |  |  |  |
|  |  |  |  |  |

Table S41: PCA Component Scores, patients with coronary artery disease

|  | **Estimate** | **Standard Error** | **p value** |  |
| --- | --- | --- | --- | --- |
| **Low Income** | 0.075 | 0.078 | 0.338 |  |
| **No High School** | -0.027 | 0.078 | 0.731 |  |
|  |  |  |  |  |
| **R-squared** | 0.00609 |  |  |  |
| **Adjusted R-squared** | 0.001326 |  |  |  |
|  |  |  |  |  |

Table S42: PCA Component Scores, patients with COPD

|  | **Estimate** | **Standard Error** | **p value** |  |
| --- | --- | --- | --- | --- |
| **Low Income** | 0.066 | 0.072 | 0.356 |  |
| **No High School** | 0.072 | 0.071 | 0.310 |  |
|  |  |  |  |  |
| **R-squared** | 0.002739 |  |  |  |
| **Adjusted R-squared** | -0.000160 |  |  |  |

Table S43: PCA Component Scores, patients with liver disease

|  | **Estimate** | **Standard Error** | **p value** |  |
| --- | --- | --- | --- | --- |
| **Low Income** | 0.107 | 0.162 | 0.51 |  |
| **No High School** | -0.199 | 0.167 | 0.234 |  |
|  |  |  |  |  |
| **R-squared** | 0.013 |  |  |  |
| **Adjusted R-squared** | -0.001200 |  |  |  |

Table S44: PCA Component Scores, patients with obesity

|  | **Estimate** | **Standard Error** | **p value** |  |
| --- | --- | --- | --- | --- |
| **Low Income** | 0.129 | 0.049 | 0.0091 | ** |
| **No High School** | 0.012 | 0.049 | 0.810 |  |
|  |  |  |  |  |
| **R-squared** | 0.02392 |  |  |  |
| **Adjusted R-squared** | 0.017040 |  |  |  |
|  |  |  |  |  |

Table S45: PCA Component Scores, patients with pulmonary disease

|  | **Estimate** | **Standard Error** | **p value** |  |
| --- | --- | --- | --- | --- |
| **Low Income** | 0.010 | 0.176 | 0.861 |  |
| **No High School** | 0.003 | 0.048 | 0.962 |  |
|  |  |  |  |  |
| **R-squared** | 0 |  |  |  |
| **Adjusted R-squared** | -0.001750 |  |  |  |
|  |  |  |  |  |

Table S46: PCA Component Scores, patients with valvular heart disease

|  | **Estimate** | **Standard Error** | **p value** |  |
| --- | --- | --- | --- | --- |
| **Low Income** | 0.372 | 0.126 | 0.0037 | ** |
| **No High School** | 0.054 | 0.126 | 0.668 |  |
|  |  |  |  |  |
| **R-squared** | 0.0688 |  |  |  |
| **Adjusted R-squared** | 0.053400 |  |  |  |
|  |  |  |  |  |

Table S47: Linear Regression Estimates, female patients

|  | **Estimate** | **Standard Error** | **p value** |  |
| --- | --- | --- | --- | --- |
| **Low Income** | 0.025 | 0.017 | 0.135 | * |
| **No High School** | 0.041 | 0.017 | 0.013 | ** |
| **Congestive Heart Failure** | 0.137 | 0.049 | 0.005 |  |
| **Valvular Disease** | 0.495 | 0.061 | 0.000 | *** |
| **Hypertension** | -0.062 | 0.038 | 0.105 |  |
| **Diabetes Mellitus** | 0.045 | 0.040 | 0.257 | *** |
| **Renal Disease** | 0.819 | 0.044 | 0.000 | *** |
| **Liver Disease** | 0.620 | 0.063 | 0.000 | *** |
| **Chronic Obstructive Pulmonary Disease** | 0.250 | 0.046 | 0.000 | *** |
| **Atrial Fibrillation** | -0.013 | 0.060 | 0.826 |  |
| **Dyslipidemia** | -0.265 | 0.042 | 0.000 | *** |
| **Coronary Artery Disease** | 0.051 | 0.048 | 0.286 | *** |
|  |  |  |  |  |
| **R-squared** | 0.05039 |  |  |  |
| **Adjusted R-squared** | 0.04969 |  |  |  |

Table S48: Linear Regression Estimates, male patients

|  | **Estimate** | **Standard Error** | **p value** |  |
| --- | --- | --- | --- | --- |
| **Low Income** | -0.060 | 0.022 | 0.006 | * |
| **No High School** | 0.107 | 0.021 | 0.000 | ** |
| **Congestive Heart Failure** | 0.148 | 0.058 | 0.011 |  |
| **Valvular Disease** | 0.478 | 0.077 | 0.000 | *** |
| **Hypertension** | 0.027 | 0.048 | 0.583 |  |
| **Diabetes Mellitus** | 0.171 | 0.051 | 0.001 | *** |
| **Renal Disease** | 0.660 | 0.051 | 0.000 | *** |
| **Liver Disease** | 0.777 | 0.069 | 0.000 | *** |
| **Chronic Obstructive Pulmonary Disease** | 0.484 | 0.058 | 0.000 | *** |
| **Atrial Fibrillation** | 0.338 | 0.063 | 0.000 |  |
| **Dyslipidemia** | -0.293 | 0.052 | 0.000 | *** |
| **Coronary Artery Disease** | -0.070 | 0.055 | 0.210 | *** |
|  |  |  |  |  |
| **R-squared** | 0.06559 |  |  |  |
| **Adjusted R-squared** | 0.06457 |  |  |  |
